# A Systematic Prospective Comparison of Fluid Volume Evaluation across OCT Devices Used in Clinical Practice

**DOI:** 10.1016/j.xops.2023.100456

**Published:** 2023-12-15

**Authors:** Klaudia Kostolna, Gregor S. Reiter, Sophie Frank, Leonard M. Coulibaly, Philipp Fuchs, Veronika Röggla, Markus Gumpinger, Gabriel P. Leitner Barrios, Virginia Mares, Hrvoje Bogunovic, Ursula Schmidt-Erfurth

**Affiliations:** 1 Department of Ophthalmology, Medical University of Vienna, Vienna, Austria; 2 Christian Doppler Laboratory for Artificial Intelligence in Retina, Department of Ophthalmology, Medical University Vienna, Vienna, Austria; 3 Department of Ophthalmology, Federal University of Minas Gerais, Belo Horizonte, Brazil

**Keywords:** Intraretinal fluid, Neovascular age-related macular degeneration, Optical coherence tomography, Pigment epithelial detachment, Subretinal fluid

## Abstract

**Objective:**

Treatment decisions in neovascular age-related macular degeneration (nAMD) are mainly based on subjective evaluation of OCT. The purpose of this cross-sectional study was to provide a comparison of qualitative and quantitative differences between OCT devices in a systematic manner.

**Design:**

Prospective, cross-sectional study.

**Subjects:**

One hundred sixty OCT volumes, 40 eyes of 40 patients with nAMD.

**Methods:**

Patients from clinical practice were imaged with 4 different OCT devices during one visit: (1) Spectralis Heidelberg; (2) Cirrus; (3) Topcon Maestro2; and (4) Topcon Triton. Intraretinal fluid (IRF), subretinal fluid (SRF), and pigment epithelial detachment (PED) were manually annotated in all cubes by trained human experts to establish fluid measurements based on expert-reader annotations. Intraretinal fluid, SRF, and PED volume were quantified in nanoliters (nL). Bland–Altman plots were created to analyze the agreement of measurements in the central 1 and 6 mm. The Friedman test was performed to test for significant differences in the central 1, 3, and 6 mm.

**Main Outcome Measures:**

Intraretinal fluid, SRF, and PED volume.

**Results:**

In the central 6 mm, there was a trend toward higher IRF and PED volumes in Spectralis images compared with the other devices and no differences in SRF volume. In the central 1 mm, the standard deviation of the differences ranged from ± 3 nL to ± 6 nL for IRF, from ± 3 nL to ± 4 nL for SRF, and from ± 7 nL to ± 10 nL for PED in all pairwise comparisons. Manually annotated IRF and SRF volumes showed no significant differences in the central 1 mm.

**Conclusions:**

Fluid volume quantification achieved excellent reliability in all 3 retinal compartments on images obtained from 4 OCT devices, particularly for clinically relevant IRF and SRF values. Although fluid volume quantification is reliable in all 4 OCT devices, switching OCT devices might lead to deviating fluid volume measurements with higher agreement in the central 1 mm compared with the central 6 mm, with highest agreement for SRF volume in the central 1 mm. Understanding device-dependent differences is essential for expanding the interpretation and implementation of pixel-wise fluid volume measurements in clinical practice and in clinical trials.

**Financial Disclosure(s):**

Proprietary or commercial disclosure may be found in the Footnotes and Disclosures at the end of this article.

Neovascular age-related macular degeneration (nAMD) is a chronic disease that is characterized by fluid accumulation in the intraretinal space (intraretinal fluid [IRF]), subretinal space (subretinal fluid [SRF]) and below the retinal pigment epithelium (RPE), referred to as pigment epithelial detachment (PED).^1^ Intravitreal anti-VEGF therapy is the gold standard treatment for nAMD, but it requires regular monitoring of disease activity and shows inferior success in patient care in the clinical setting compared with clinical studies.^2^, ^3^, ^4^, ^5^ With a growing elderly population, optimization of age-related macular degeneration management would relieve a health care system overburdened with an estimated global prevalence of 170 million patients with this condition, expected to rise to 288 million by the year 2040.^6^^,^^7^ Furthermore, upcoming therapies in exudative and nonexudative macular disease will increase the burden on hospitals and the demand for automated support systems.^8^^,^^9^ Consequently, reliable, high-quality diagnostic devices and precise biomarker assessment are essential for timely disease detection and personalized treatment decisions.^10^, ^11^, ^12^

OCT is the most powerful, noninvasive modality for imaging the retina.^13^^,^^14^ The hardware and software of OCT devices have rapidly evolved since its introduction for ocular axial length measurements in 1988.^15^ The progress from time-domain to Fourier-domain imaging technology increased the scanning speed and enabled higher B-scan rates, resulting in 3-dimensional volume images.^16^ This crucial step led to a higher consensus in clinical interpretation and faster detection of disease activity.^16^, ^17^, ^18^ Hence, study end points and treatment decisions in clinical practice are routinely based on macular structure analyses on swept-source (SS)-OCT and spectral domain (SD)-OCT devices.^10^^,^^13^^,^^14^^,^^17^ Swept-source-OCT uses longer center wavelengths for faster acquisition speed and deeper light penetration into the eye and therefore has optimized choroidal visualization with reduced axial resolution compared with SD-OCT.^19^ Clinical trials and OCT analyses in the current literature frequently encompass SD-OCT manufacturers Zeiss, Heidelberg, and Topcon.^20^ Concurrently, the combination of the volumetric display of retinal morphology with OCT angiography on SS-OCT systems are broadly applied in clinic and examined throughout the literature.^1^^,^^21^

In recent years, validation of automated algorithms for OCT biomarker quantification has continuously demonstrated that artificial intelligence (AI) is able to extract and quantify information from OCT volumes on a voxel level in a fast and objective manner and performs equally to human experts.^22^, ^23^, ^24^, ^25^ Currently, deep-learning algorithms are being developed on devices from different manufacturers and are most prevalently implemented on SD-OCTs from Zeiss, Heidelberg, and Topcon,^26^ while developments on SS-OCTs, such as the Topcon Triton, are also explored.^27^ The use of different OCT systems for AI development still represents a challenge because algorithms need to be trained and validated based on device-specific characteristics for optimal performance.^28^ In the novel era of AI in the retina, personalized nAMD treatment will depend on consistent biomarker quantification throughout this major spectrum of frequently used OCTs.^29^ However, human expert annotations are the gold standard for training AI algorithms for biomarker quantification. In this study, fluid volume was quantified in 3 commonly used SD-OCTs, Zeiss Cirrus, Heidelberg Spectralis, and Topcon Maestro2, and one SS-OCT, Topcon Triton. Retinal fluid volumes were compared between these commercially available devices based on human expertise. The comparison of fluid volume measurements throughout commonly used OCT systems is an essential step for expanding the application of AI algorithms in clinical practice. To date, this is the first work that compares human expert annotations of fluid in nAMD between commonly used OCT devices.

## Methods

### Patients and Device Characteristics

Forty eyes from 40 patients with nAMD that received standard-of-care treatment at the Medical University in Vienna (Austria) were included based on the diagnosis of nAMD. Fluid could only be present in one compartment, as IRF, SRF, or PED, for inclusion in this cross-sectional study. The protocol was approved by the Ethics Committee of the Medical University of Vienna (EC: 2094/2018) and adhered to tenets of the Declaration of Helsinki. All patients gave written informed consent before enrollment. Diagnosis of nAMD was clinically determined by a retina specialist via slit lamp examination, OCT, OCT angiography, and/or fluorescein angiography. All 40 patients underwent OCT imaging with 4 different OCT devices (3 SD-OCT and 1 SS-OCT) during a single visit: (1) SD-OCT Spectralis Heidelberg HRA + OCT (Spectralis, Heidelberg Engineering GmbH, serial number [sn]: Spec-KT-05432, manufacture date [md]: 2015-02 or sn: Spec-KT-09262, md: 2021-03); (2) SD-OCT Cirrus HD-OCT (Cirrus, Carl Zeiss Meditec, model number: Cirrus 5000, sn: 5000-4287, md: 2014-05 or model number: Cirrus 4000, sn: 4000-1654, md: 2008-02); (3) SD-OCT Topcon 3D OCT- 1 Maestro2 (Maestro, sn: AB9002032, md: 2020-10); and (4) SS-OCT Topcon Triton DRI OCT (Triton, sn: 991356, md: 2021-05 both by Topcon, Tokyo, Japan). The image settings and technical properties of each device are summarized in Table 1.

**Table 1:** Image Settings and Technical Differences in All 4 OCT Devices

Device	Wavelength	A-scan/s A-scan/B-scan	Axial Resolution	Macular Cube	Number of B-scans
SPECTRALIS∗	870 nm	85 000 /s High-Resolution: 1024/ B-scan	< 7 μm	6 × 6 mm	97
CIRRUS∗	840 nm	27 000 /s 512/ B-scan	5 μm	6 × 6 mm	128
MAESTRO∗	840 nm	50 000/s 512/ B-scan	6 μm	6 × 6 mm	128
TRITON†	1050 nm	100 000/s 512/ B-scan	8 μm	7 × 7 mm	256

Cirrus = Cirrus HD-OCT (Carl Zeiss Meditec, Inc); HR = high resolution; Maestro = Topcon 3D OCT- 1 Maestro2 (Topcon); Spectralis = Spectralis Heidelberg HRA + OCT (Heidelberg Engineering); Triton = Topcon Triton DRI OCT (Topcon).

∗ Spectral domain-OCT.

† Swept-source OCT.

### Image Analysis

#### Retinal Fluid Volume Evaluation

**Figure 1:**
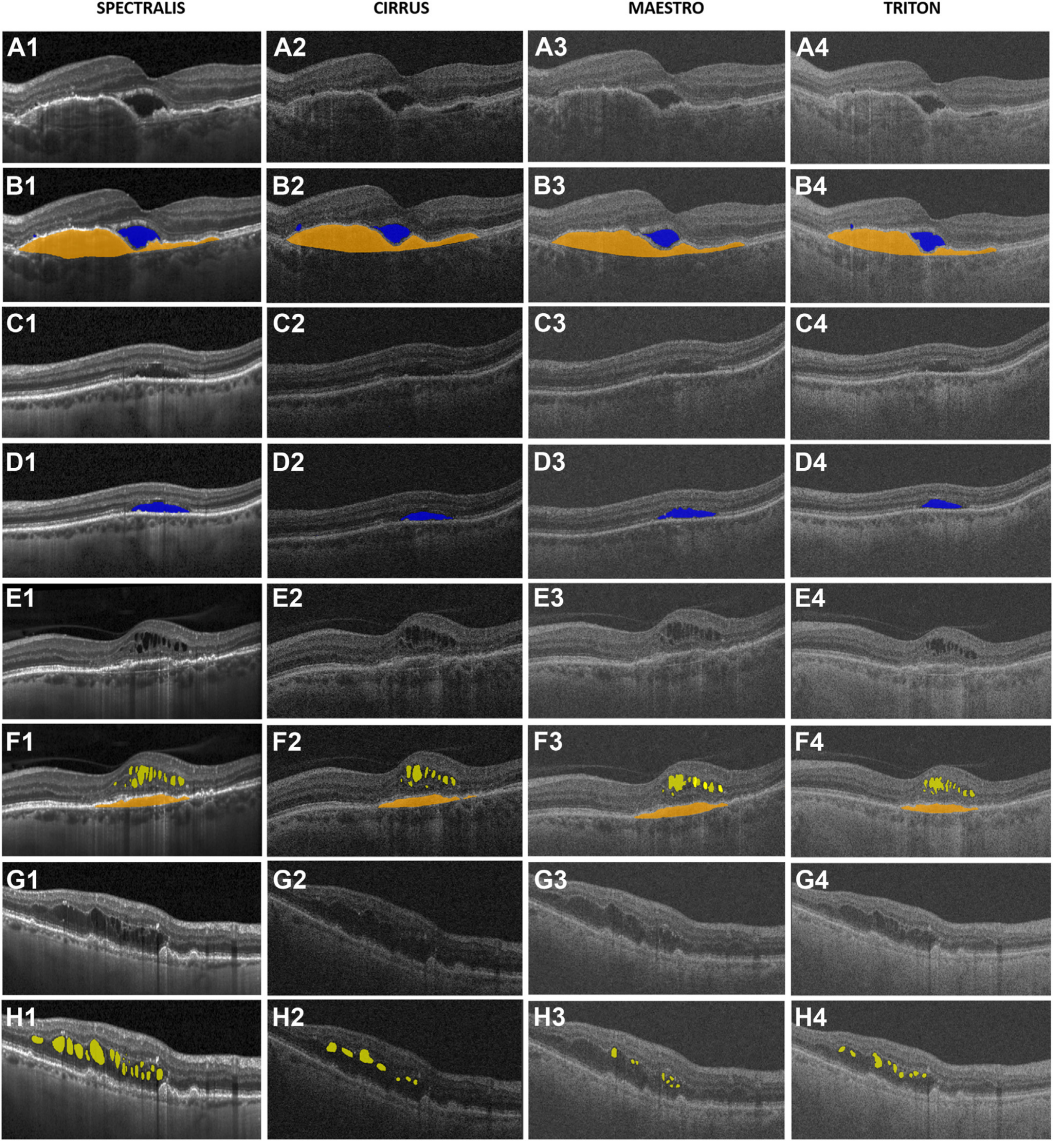
Pixel-wise expert reader intraretinal fluid (IRF), subretinal fluid (SRF), and pigment epithelial detachment annotations. **A**, Inner border of SRF easily distinguishable in all 4 OCT scans. **B**, Pixel-wise annotations with very similar labels in all 4 devices. **C**, Inner borders of SRF are hard to distinguish on Cirrus and Maestro but clearly delineated in Spectralis. **D**, Pixel-wise annotations are similar on all 4 devices despite qualitative differences. **E**, The IRF borders are delineated in all 4 OCT devices. **F**, Pixel-wise annotations with very similar labels in all 4 devices. **G**, The IRF borders are delineated in Spectralis but hard to distinguish in Cirrus, Maestro, and Triton. **H**, Pixel-wise annotations show qualitative differences.

An AI-based algorithm automatically segmented the fluid compartments with each voxel classified by a multiscale convolutional neural network. In short, this convolutional neural network applies deep-learning to map OCT images to pixel-level class labels based on large amounts of labeled training data. Sematic segmentation allows the neural network to map an input image of a specific size to an image of class labels of the same size. This is based on an encoder that transforms an input image into an abstract representation and a decoder that maps the abstract representation to an image of clinical class labels. Therefore, each pixel is assigned the label IRF, SRF, or PED or healthy tissue.^30^ Pigment epithelial detachment was segmented based on a previously validated algorithm that segments the region between the RPE and Bruch’s membrane (BM).^31^ The algorithm was trained and validated as described previously.^30^^,^^31^ Manual pixel-wise corrections of the AI-based segmentation of IRF, SRF, and PED were performed by an expert reader (K.K.) trained according to reading center standards to ensure the comparison of these devices based on human expertise and avoid comparison of algorithm performance on the specific device, as deep-learning algorithms are not yet validated for all 4 devices used in this study. Difficult cases were discussed in a group with senior retina specialists (V.M. and G.R.) until consensus was reached. The reader was masked to the segmentations on the other devices and performed all gradings independently for each device. In the manual grading protocol, IRF was defined as distinct hyporeflective regions within the neurosensory retina, including all layers between the internal limiting membrane and the ellipsoid zone. Subretinal fluid was defined as a hyporeflective space between the ellipsoid zone and the RPE. Pigment epithelial detachment was defined as an elevation of the RPE from BM with fibrovascular and/or serous components. The threshold for minimum PED width was set at 300 μm, which is 50 μm narrower than previously defined to avoid identification of borderline PEDs, if present.^1^ There was no threshold for PED height. Once a defined PED was marked, annotations of the same lesion were continued in adjacent B-scans regardless of the lesion size. Figure 1 demonstrates examples of manual pixel-wise annotations for each device.

Corrections were conducted in the total macular OCT volume consisting of 97 B-scans (3880 B-scans in total) in Spectralis. In Cirrus and Maestro, every second B-scan was annotated, manually correcting 64 B-scans per OCT volume (2560 B-scans/device in total). For Triton, the reader corrected every fourth B-scan, manually marking 64 B-scans per OCT volume (2560 B-scans in total). Fluid volume measurements were only calculated in manually corrected B-scans. B-scans that were not corrected were removed from the measurements because it has been demonstrated previously that there is no significant difference in fluid volume calculations between 64 B-scans and 97 B-scans.^32^ The IRF, SRF, and PED volumes were computed in the common Early Treatment of Diabetic Retinopathy Study macular grid in the central 1, 3, and 6 mm and analyzed in nanoliters (nL). The IRF, SRF, and PED volumes were summed to determine the total fluid volume (TFV) for each OCT volume. The position of the fovea was set manually in each OCT volume as a reference point for volume comparison.

### Statistical Analysis

This is an explorative data analysis. Descriptive statistics were calculated for each retinal fluid compartment in the central 1 and 6 mm. Bland–Altmann plots were created to analyze the limits of measurement agreement and the presence of systematic bias between 2 devices separately for all 3 fluid compartments in the central 1 and 6 mm. For SRF, the agreement of fluid measurements was additionally examined within a 10-nL threshold in the central 1 mm because SRF-tolerating regimes have been discussed in the recent literature.^12^^,^^33^

The Friedman test, a nonparametric test for dependent samples with post hoc pairwise comparisons using Bonferroni correction, was performed to test for significant differences in IRF, SRF, PED, and TFV between all 4 devices in the central 1, 3, and 6 mm. Intraclass correlation coefficients (ICCs) and their 95% confidence intervals (CIs) were calculated based on a mean-rating (k = 4), consistency, 2-way mixed-effects model. The data were analyzed with SPSS statistical software. The alpha error was set to *P* < 0.05.

## Results

A total of 160 OCT volumes from 40 eyes of 40 patients with 11 560 corrected B-scans were analyzed. Twenty-four patients (60%) were female, and 40% were male. The mean patient age was 78.85 ± 7.3 years. Descriptive statistics for IRF, SRF, and PED in the central 1 and 6 mm are summarized in Table 2.

**Table 2:** Descriptive Statistics from the Central 1 and 6 mm

Device	IRF		SRF		PED	
	1 mm	6 mm	1 mm	6 mm	1 mm	6 mm
*Spectralis n*	n = 22	n = 31	n = 23	n = 25	n = 40	n = 40
*Cirrus n*	n = 24	n = 27	n =24	n = 25	n = 39	n = 40
*Maestro n*	n = 24	n = 30	n = 22	n = 25	n = 38	n = 40
*Triton n*	n = 22	n =32	n = 22	n = 24	n = 37	n = 40
	Median (nL)∗ Min.–Max.(nL)		Median (nL)∗ Min.–Max.(nL)		Median (nL)∗ Min.–Max.(nL)	
*Spectralis nL*	11 nL *0.1–86 nL*	19 nL *0.8–277 nL*	7 nL *0.0–110 nL*	36 nL *0.3–2193 nL*	31 nL *0.5–270 nL*	262 nL *21–3162 nL*
*Cirrus nL*	4 nL *0.1–67 nL*	22 nL *2–179 nL*	5 nL *0.1–105 nL*	36 nL *0.2–2510 nL*	31 nL *0.1–294 nL*	207 nL *15–2922 nL*
*Maestro nL*	4 nL *0.1–77 nL*	24 nL *0.5–170 nL*	10 nL *0.2–102 nL*	45 nL *0.2–1872 nL*	31 nL *1.4–253 nL*	249 nL *22–2879 nL*
*Triton nL*	9 nL *0.1–81nL*	20 nL *0.4–177 nL*	8 nL *0.1–89 nL*	37 nL *0.3–2204 nL*	34 nL *0.6–269 nL*	219 nL *6–2868 nL*
	ICC		ICC		ICC	
*Spectralis-Cirrus-Maestro-Triton*	0.988 (CI 0.980–0.993)	0.939 (CI 0.900–0.965)	0.996 (CI 0.993–0.998)	0.996 (CI 0.994–0.998)	0.998 (CI 0.996–0.999)	0.997 (CI 0.996–0.999)

Varying fluid presence dependent on the device concerned only volumes below 2 nL. CI = 95% confidence interval; Cirrus = Cirrus HD-OCT (Carl Zeiss Meditec, Inc.); ICC = intraclass correlation coefficient; IRF = intraretinal fluid; Maestro = Topcon 3D OCT- 1 Maestro2 (Topcon); Max. = maximum; Min. = minimum; PED = pigment epithelial detachment; SRF = subretinal fluid; Triton = Topcon Triton DRI OCT (Topcon); 1 mm = central 1 mm; 6 mm = central 6 mm.

∗ Median for number of patients with IRF, SRF, and PED present > 0 nL.

### Qualitative Differences in Fluid Volumes

Figure 1 demonstrates the qualitative differences of all 4 OCT devices with pixel-wise human expert annotations. Figure 1B, F shows examples of clearly delineated IRF and SRF borders in all OCT devices with very similar pixel-wise expert reader gradings. Figure 1D, H shows examples with unclear IRF and SRF borders with differences in pixel-wise expert reader annotations, especially for IRF (Fig 1H). The B-scans from different devices vary in their signal-to-noise ratio, axial resolution, reflectivity, and contrast. Spectralis’ B-scan averaging and higher signal-to-noise ratio facilitated the recognition of the 360° IRF borders, the ellipsoid zone as the SRF border, and BM as the PED border in challenging cases.

### Quantitative Variability in Fluid Volumes

**Figure 2:**
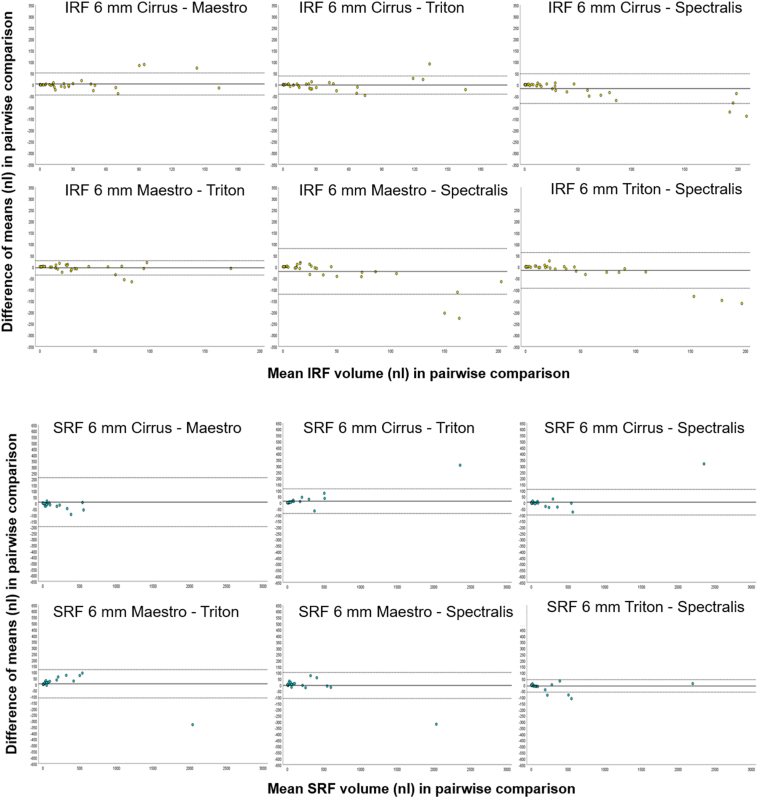
Bland–Altman plots of intraretinal fluid (IRF) volume and subretinal fluid (SRF) volume in all 6 pairwise comparisons between 2 devices separately (yellow and blue dots). The 95% limits of agreement (mean difference ± 1.96 standard deviation of the difference) are plotted with dashed lines. Cirrus = Cirrus HD-OCT (Carl Zeiss Meditec, Inc.); Maestro = Topcon 3D OCT- 1 Maestro2 (Topcon); Spectralis = Spectralis Heidelberg HRA + OCT (Heidelberg Engineering); Triton = Topcon Triton DRI OCT (Topcon).

**Figure 3:**
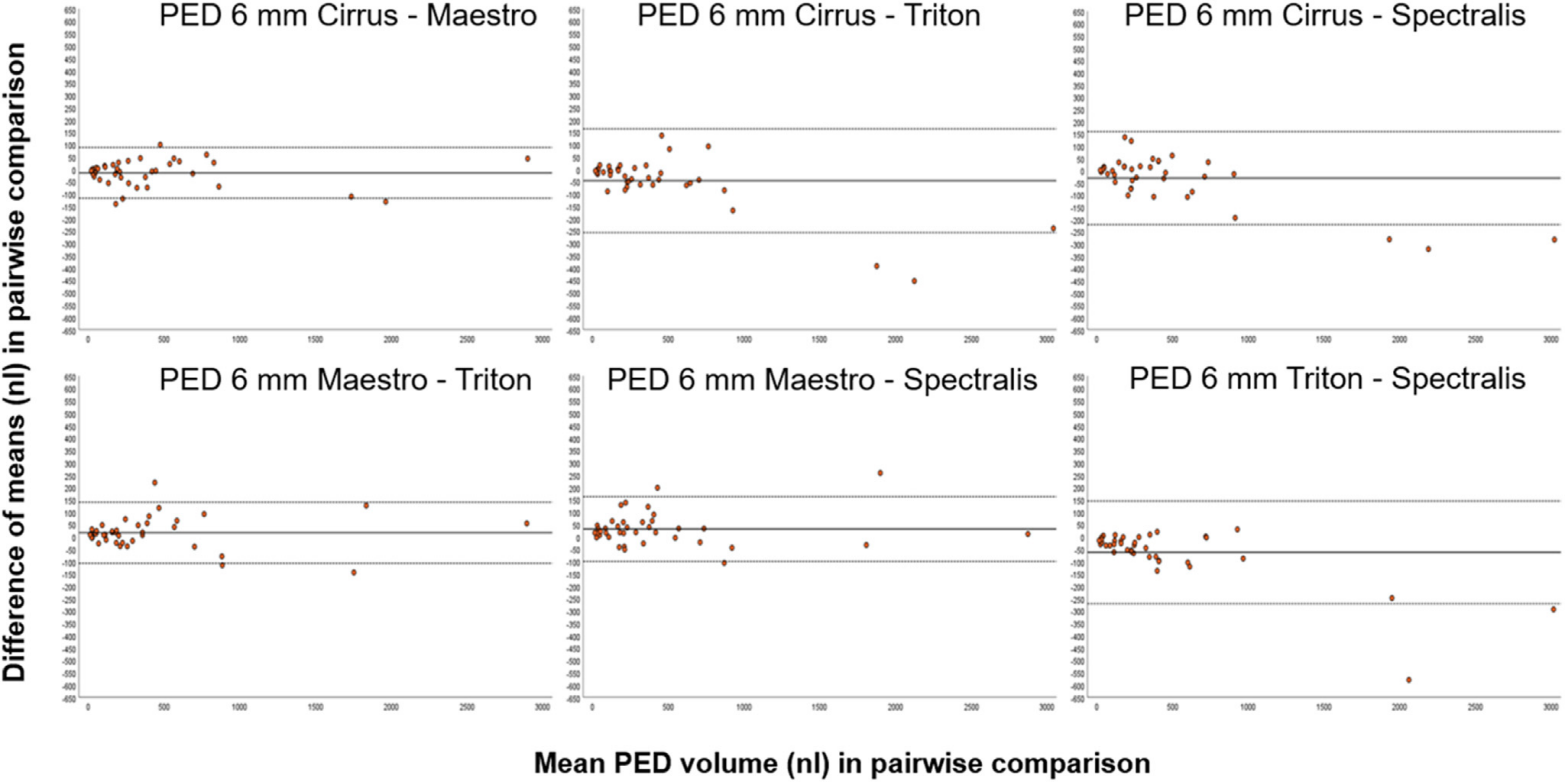
Bland–Altman plots of pigment epithelial detachment (PED) volume in all 6 pairwise comparisons between 2 devices separately (orange dots). The 95% limits of agreement (mean difference ± 1.96 standard deviation of the difference) are plotted with dashed lines. Cirrus = Cirrus HD-OCT (Carl Zeiss Meditec, Inc.); Maestro = Topcon 3D OCT- 1 Maestro2 (Topcon); Spectralis = Spectralis Heidelberg HRA + OCT (Heidelberg Engineering); Triton = Topcon Triton DRI OCT (Topcon).

In the central 1 mm, IRF, SRF, and PED volume measurements achieved excellent reliability between all 4 devices with an ICC of 0.988 (95% CI, 0.980–0.993), 0.996 (95% CI, 0.993–0.998), and 0.998 (95% CI, 0.996–0.999), respectively (Table 2). In the central 6 mm, IRF, SRF, and PED volumes showed also excellent reliability between all 4 devices with an ICC of 0.939 (95% CI, 0.900–0.965), 0.996 (95% CI, 0.994–0.998), and 0.997 (95% CI, 0.996–0.999), respectively (Table 2). Figures 2 and 3 display the Bland–Altman plots for IRF, SRF, and PED volume in the central 6 mm.

### Evaluation in the Wider 6-mm Area

The results from all pairwise comparisons in the central 6 mm for all fluid compartments are summarized in Table 3, including the 95% limits of measurement agreement, standard deviation (SD) of differences, and difference of means (dM). There was a trend toward higher IRF volume measurements in Spectralis compared with Maestro, Cirrus, and Triton, as graphically displayed in Figure 2 for each device comparison separately. The agreement within the limits of measurement agreement was lower in higher IRF volumes in the central 6 mm. The highest dM was calculated between Maestro and Spectralis (–20 nL, SD ± 51 nL), followed by Cirrus and Spectralis (–17 nL, SD ± 33 nL) and Triton and Spectralis (–15 nL, SD ± 40 nL). For SRF, there was no trend or bias in any of the pairwise comparisons (Table 3), whereas PED volume showed high differences in all pairwise comparisons with a trend toward higher volume measurements in Spectralis compared with the 3 other devices (Fig 3). For PED, the highest dM was measured between Triton and Spectralis (–63 nL, SD ± 106 nL), followed by Cirrus and Spectralis (–47 nL, SD ± 107 nL), Maestro and Spectralis (–32 nL, SD ± 97 nL), and the lowest between Cirrus and Maestro (dM –15 nL, SD ± 53).

**Table 3:** 95% Limits of Agreement from the Bland–Altman Plots, Standard Deviation of Differences, Difference of Means, and in the Central 1 mm and 6 mm in all 6 Pairwise Comparisons

Device Comparison	1 mm			6 mm		
	95% limits B-A-plots			95% limits B-A-plots		
	IRF	SRF	PED	IRF	SRF∗	PED
CIRRUS-MAESTRO	+12 nL –12 nL	+6 nL –8 nL	+18 nL –15 nL	+51 nL –45 nL	+212 nL –196 nL	+88 nL –118 nL
CIRRUS-TRITON	+12 nL –13 nL	+9 nL –7 nL	+17 nL –12 nL	+38 nL –41 nL	+113 nL –88 nL	+140 nL –107 nL
CIRRUS-SPECTRALIS	+9 nL –13 nL	+5 nL –6 nL	+19 nL –20 nL	+48 nL –82 nL	+108 nL –101 nL	+164 nL –257 nL
MAESTRO-SPECTRALIS	+7 nL –11 nL	+7 nL –6 nL	+14 nL –19 nL	+80 nL –120 nL	+102 nL –110 nL	+158 nL –222 nL
MAESTRO-TRITON	+5 nL –6 nL	+6 nL –4 nL	+14 nL –12 nL	+27 nL –36 nL	+121 nL –112 nL	+163 nL –100 nL
TRITON-SPECTRALIS	+6 nL –9 nL	+8 nL –9 nL	+14 nL –20 nL	+63 nL –94 nL	+43 nL –60 nL	+145 nL –271 nL

	1 mm			6 mm		
	Standard deviation of differences			Standard deviation of differences		
	IRF	SRF	PED	IRF	SRF	PED
CIRRUS - MAESTRO	± 6 nL	± 3 nL	± 8 nL	± 25 nL	± 104 nL	± 53 nL
CIRRUS-TRITON	± 6 nL	± 4 nL	± 7 nL	± 20 nL	± 51 nL	± 63 nL
CIRRUS-SPECTRALIS	± 6 nL	± 3 nL	± 10 nL	± 33 nL	± 53 nL	± 107 nL
MAESTRO-SPECTRALIS	± 5 nL	± 4 nL	± 8 nL	± 51 nL	± 54 nL	± 97 nL
MAESTRO-TRITON	± 3 nL	± 3 nL	± 7 nL	± 16 nL	± 59 nL	± 67 nL
TRITON-SPECTRALIS	± 4 nL	± 4 nL	± 9 nL	± 40 nL	± 26 nL	± 106 nL

	1 mm			6 mm		
	Differences of means			Differences of means		
	IRF	SRF	PED	IRF	SRF	PED
CIRRUS-MAESTRO	–0.2 nL	–0.8 nL	2 nL	–3 nL	8 nL	–15 nL
CIRRUS-TRITON	–0.7 nL	0.6 nL	2 nL	–2 nL	12 nL	16 nL
CIRRUS-SPECTRALIS	–2 nL	–0.2 nL	–0.7 nL	–17 nL	4 nL	–47 nL
MAESTRO-SPECTRALIS	–2 nL	0.6 nL	–2 nL	–20 nL	–4 nL	–32 nL
MAESTRO-TRITON	–0.5 nL	1 nL	0.6 nL	–5 nL	5 nL	31 nL
TRITON-SPECTRALIS	–1 nL	–0.8 nL	–3 nL	–15 nL	8 nL	–63 nL

B-A-plots = Bland–Altman plots; Cirrus = Cirrus HD-OCT (Carl Zeiss Meditec, Inc.); IRF = intraretinal fluid; Maestro = Topcon 3D OCT- 1 Maestro2 (Topcon); PED = pigment epithelial detachment; SRF = subretinal fluid; Triton = Topcon Triton DRI OCT (Topcon); 1 mm = central 1 mm; 6 mm = central 6 mm.

∗ 95% limits for SRF volume without 1 outlier: Cirrus-Maestro +30 nL, –47 nL; Cirrus-Triton +43 nL, −33 nL; Cirrus-Spectralis +28 nL, –36 nL; Maestro-Spectralis +39 nL, –30 nL; Maestro-Triton +59 nL, –33 nL; Triton-Spectralis +43 nL, –61 nL.

### Evaluation in the Central 1-mm Area

The results from all pairwise comparisons in the central 1 mm for all fluid compartments are summarized in Table 3. For IRF, the SD of the differences was between ± 3 nL and ± 6 nL with dM between 0.2 and 2 nL. For SRF, the SD of the differences were between ± 3 and ± 4 with a dM ≤ 1 nL in all pairwise comparisons. Pigment epithelial detachment had the highest SD and dM in the central 1 mm with SD of differences between ± 7 nL and ± 10 nL and dM between 0.7 nL and 3 nL.

In the pairwise comparisons, 98% (39/40) of the SRF volume differences were within the 10 nL threshold between Cirrus and Maestro, 95% (38/40) between Cirrus and Triton, 100% (40/40) between Cirrus and Spectralis, 98% (39/40) between Maestro and Triton, 98% (39/40) between Maestro and Spectralis, and 98% (39/40) between Triton and Spectralis.

### Evaluation of Differences between 1-mm and 6-mm Areas

The Friedman test showed no significant differences in IRF volume in the central 1, 3, and 6 mm.

The SRF volumes did not differ significantly in the central 1 mm. In the central 6 mm, there were significant differences in SRF volume between Triton and Spectralis (*P* = 0.026), Triton and Cirrus (*P* = 0.004), and Triton and Maestro (*P* = 0.004). In the central 3 mm, SRF volume differed significantly between Triton and Spectralis (*P* = 0.038) and Triton and Maestro (*P* = 0.002).

For PED volume in the central 1 mm, there was a significant difference between Triton and Spectralis (*P* = 0.006). In the central 6 mm, PED volume differed significantly between Triton and Spectralis (*P* < 0.001), Triton and Maestro (*P* = 0.003), and Cirrus and Spectralis (*P* = 0.034) and in the central 3 mm, between Triton and Spectralis (*P* < 0.001), Triton and Maestro (*P* = 0.015), and Cirrus and Spectralis (*P* = 0.026).

### TFV Comparison

The TFV was used as an additional outcome parameter, because not all fluid compartments were represented in each eye simultaneously. There was excellent reliability for TFV in the central 1 and 6 mm (ICC 0.997 [95% CI, 0.995–0.998] and 0.998 [95% CI, 0.997–0.999], respectively). In the central 1 mm, there were significant differences between Triton and Maestro (*P* = 0.044) and Cirrus and Spectralis (*P* < 0.001). In the central 6 mm, significant differences were found between Triton and Maestro (*P* = 0.011), Triton and Spectralis (*P* < 0.001), and Cirrus and Spectralis (*P* = 0.011). In the central 3 mm, Triton and Maestro (*P* = 0.026), Triton and Spectralis (*P* < 0.001), and Spectralis and Cirrus (*P* = 0.002) differed significantly.

### Impact of B-Scan Rate

The influence of B-scan rate on fluid volume was further examined in the Spectralis device. No statistically significant differences in TFV, IRF, SRF, and PED volume were found between 64 B-scans and 97 B-scans in the central 1, 3, and 6 mm.

## Discussion

A data set of 160 OCT volumes with 11 560 manually annotated B-scans was analyzed in this cross-sectional study. The goal of this study was to quantify retinal fluid in the frequently used SD-OCT devices and one SS-OCT device in clinical practice to establish whether IRF, SRF, and PED volumes are accurately quantifiable and comparable throughout devices.

Understanding the device-specific characteristics facilitates the interpretation of our results. Cirrus and Maestro have similar acquisition speed with the same B-scan spacing and comparable center wavelengths and axial resolution. For Cirrus, pupil position and focus have to be set by the examiner, whereas Maestro performs with a self-sufficient acquisition after the pupil position has manually been identified. The position of the macular cube cannot be moved on the Maestro device, and the quality may suffer by bad fovea centration in noncompliant patients. The only SS-OCT in this study, Triton, scans the retina faster than SD-OCT devices with the highest number of B-scans and allows for a better visualization of the choroid. In Triton, pupil position and focus are manually controlled by the examiner, whereas the position of the macular cube cannot be moved manually, which might lead to worse foveal centration as described previously. Motion artifacts are minimalized by faster acquisition speed. For Spectralis, multiple image settings can be chosen. The examiner controls the position of the macular cube, pupil position, focus, and illumination during the exam. These manual adjustments require experience and expertise and are crucial for good image quality. In the high-resolution mode with B-scan averaging set at 16 frames, motion artifacts are prevented due to B-scan averaging. However, longer acquisition time is strenuous and requires the patient’s concentration. In summary, Maestro and Cirrus are comparable devices with regard to fluid volume measurements, quality, and user experience, whereas Spectralis produces B-scans with the highest signal-to-noise ratio with easier fluid delineation. Triton is the most distinctive of the other devices due to the different size of the macular cube (7 × 7 mm compared with 6 × 6 on Spectralis, Cirrus, and Maestro), technical fundamentals with the highest B-scan rate (256 B-scans compared with 128 in Cirrus and Maestro2 and 97 B-scans in Spectralis), and acquisition speed.

Analyses of the Bland–Altman plots showed the highest agreement for SRF in the central 1 mm and a potentially clinically significant difference in IRF and PED volumes in the central 6 mm with a trend toward higher measurements in Spectralis compared with the other devices. No significant differences in IRF and SRF volume in the central 1 mm were found, whereas PED volume differed significantly between OCT devices. We demonstrated that fluid is quantifiable and comparable between Spectralis, Cirrus, and 2 Topcon OCTs with excellent reliability with ICC values above 0.94 for all fluid compartments in the central 6 mm. Total fluid volume analysis was used as an additional outcome parameter and demonstrated excellent reliability with significant differences between devices with less or more background noise (Spectralis and Cirrus) and between SS-OCT and SD-OCT (Spectralis and Triton, Maestro and Triton). For clinical outcomes in clinical practice, each fluid compartment influences the morphological and functional outcomes differently.^34^ Intraretinal fluid and SRF, compartments that trigger treatment decisions with anti-VEGF, showed no significant differences between any of the devices in the clinically significant central 1 mm. A trend toward higher IRF volume measurements in Spectralis in the central 6 mm can be explained by difficulties in clearly distinguishing IRF borders in other devices. Subretinal fluid did not exhibit any bias or trend between any of the 2 devices in the Bland–Altman plots in the central 1 mm. More importantly, SRF had the narrowest 95% limits of agreement with values under 10 nL in the central 1 mm in all pairwise SRF volume comparisons (Table 3). Additionally, 98% to 100% of SRF volume differences in all 6 pairwise comparisons were within a 10-nL threshold in the central 1 mm. This finding is of high relevance, as SRF-tolerating treatment regimens are currently discussed controversially.^12^^,^^33^ Currently, there is no evidence on clinically relevant thresholds for fluid volumes. However, thresholds are being examined throughout the literature on automated fluid quantification,^12^^,^^35^ similar to threshold for central retinal thickness (CRT) in current treatment regimes.^36^ The threshold of 10 nL in the central 1 mm cannot be translated yet to clinical practice but allows for a more thorough understanding of the data analyzed in this paper, and therefore, it broadens our understanding on fluid volume quantification. We postulate that differences in SRF volume in the central 6 mm between Triton and Spectralis, Cirrus, and Maestro are due to the very distinctive imaging pattern of the SS-OCT, Triton, with the most deviating B-scan spacing and a different imaging area. Nevertheless, we conclude that SRF volume is the fluid compartment with the highest agreement between different OCT manufacturers, which is reflected in the 95% limits of agreement from the Bland–Altman plots in the central 1 and the 6 mm after one single outlier correction (Table 3). Pigment epithelial detachment presence or PED volume generally does not influence treatment decisions in clinical practice. However, PED volume impacts visual acuity^37^ and differed significantly between OCTs from various manufacturers in this analysis. Analyses of the Bland–Altman plots demonstrated that the highest volume differences were found in PED measurements in the central 1 and 6 mm (Table 3). Differences in the identification of BM, which might lead to overcorrection or undercorrection of PEDs in different OCT devices, lead to these volume differences, whereas the recognition of BM is easier with less background noise. Additionally, as PEDs were marked in almost each B-scan according to our annotation protocol, minimal deviations of the corrected anatomical region might result in differences in the calculated PED volume.

To date, the only established quantitative biomarkers on OCT are CRT and central subfield thickness, which show weak correlations with visual acuity in nAMD.^38^^,^^39^ Substantial differences in CRT and central subfield thickness in different OCT devices have been reported by several groups.^40^^,^^41^ Furthermore, the highest variability in retinal thickness occurs in areas most affected by macular edema.^40^ The boundaries of retinal layers in the automated CRT software differ between Spectralis, Cirrus, and Topcon OCT devices and measure significantly different central subfield thickness values in nAMD.^41^ Thus, automated CRT measurements from device-dependent software cannot be used interchangeably between different OCTs without manual readjustments.^42^^,^^43^

Our findings are of high relevance not only for multicenter clinical trials with imaging protocols on different OCT devices but also for treatment routine in nAMD. Several research groups are developing and implementing AI-based fluid quantification on different OCT devices in data sets from nAMD patients from clinical settings and trials.^25^^,^^30^^,^^37^ Additionally, personalized treatment with AI-based fluid quantification is underway in clinical practice as a decision support.^12^^,^^44^ Early findings suggest that fluid volume is a precise and objective biomarker that allows for individual treatment monitoring of nAMD activity with lower levels of fluid volume being associated with superior visual outcomes.^34^ In the real world, varying acquisition protocols, background noise, and gray scales of fluid are challenges in the unification of AI algorithm performance.^45^ Fluid volume quantification in the clinic can only be based on automated algorithms, because manual IRF, SRF, and PED delineation in OCT B-scans is not applicable to busy clinical practice. Currently, most AI algorithms for automated fluid segmentation are trained based on manually annotated human expert reader data sets. Therefore, it is of utmost importance to analyze and define the fluid volume differences between commonly used OCT machines based on human expertise. Based on our results and previous work from the literature, we postulate that fluid quantification is dependent on proper fovea centration, B-scan spacing, and signal-to-noise ratio of the respective device used. Lower IRF fluid volume in the central 6 mm is measured in devices with lower signal-to-noise ratio compared with higher signal-to-noise ratio. Because IRF has been proven to be a fluid compartment with the highest impact on anatomical and functional outcomes,^34^ clinicians should consider these device-dependent changes in IRF volume.

Strengths of this analysis include the large study cohort imaged with multiple devices in the same day visit and optimal expert reader annotations of all compartments. Moreover, we present the first quantitative comparison of nAMD fluid biomarkers in different OCTs based on trained reader expertise in the 4 most frequently used OCT devices. Artificial intelligence combined with manual annotations by human graders provides the most robust evidence.

This study has limitations that should be considered when interpreting the data. First, analyses of the limits of agreement in the Bland–Altman plots in a cohort of 40 patients may lead to misinterpretation, as limits of agreement are calculated based on the SD of the differences between 2 devices. Therefore, outliers have a great impact on the calculated limits of agreement. Removing these outliers from the measurements would not mirror the reality in clinic. A post hoc analysis of the limits of agreement without this one particular outlier are summarized in the legend of Table 3. These results are closer to the real differences in SRF volume in the central 6 mm, in our opinion.

Second, the spacing of every second (Maestro, Cirrus) and every fourth (Triton) B-scan from the top to the bottom of the macular cube was chosen to compare the volume in the same number of B-scans in these 3 devices because the scanning density is 2 times higher in Triton than in Maestro and Cirrus. Consequently, minimal deviations in the position of the macular cube have an impact on the anatomical region that is analyzed in one single B-scan. Lack of an interdevice follow-up function means that the position of the macula cube might deviate minimally between the devices. However, previous studies demonstrated that no statistically significant differences in fluid volume are observed between 128 and 64 B-scans, and a minimum of 16 B-scans is sufficient to generate comparable volume maps.^32^ Considering that pixel-wise manual corrections of one OCT volume require between 3 to 8 hours for experienced readers, annotating each B-scan with 256 B-scans (Triton) and 128 B-scans (Maestro, Cirrus) would decrease the feasibility of these analyses without adding any pivotal value. The impact of B-scan density in this study cohort was further investigated in our subanalysis in Spectralis. No significant differences were found between Spectralis 64 B-scans and Spectralis 97 B-scans. However, with the prospect of applying these results to clinical practice, the standardized B-scan spacing in this study needs to be considered. With AI implementation to clinic on each device, clinicians should be aware of the fact that narrower B-scan spacing could influence fluid volume calculations, as AI-based fluid segmentation in clinical practice is not standardized and performed on each available B-scan.^23^ The third limitation is that although manual grading was performed with certified human expertise, a subjective aspect is inevitable in difficult cases. Such subjectivity would be reduced by reliable automated tools for each OCT device.

In conclusion, although fluid volume quantification is reliable in all 4 OCT devices, switching OCT devices might lead to different fluid volume measurements. However, there may be higher agreement in the central 1 mm compared with the central 6 mm, as summarized for each retinal fluid compartment. Understanding device-dependent fluid volume differences is essential for expanding the implementation and interpretation of AI-based fluid quantification in clinical trials and practice.
